# Advancements in Marburg (MARV) Virus Vaccine Research With Its Recent Reemergence in Equatorial Guinea and Tanzania: A Scoping Review

**DOI:** 10.7759/cureus.42014

**Published:** 2023-07-17

**Authors:** Manish P Mane Manohar, Vivian J Lee, Ejikeme U Chinedum Odunukwe, Pratik K Singh, Buhlebethu S Mpofu, Christine Oxley, MD

**Affiliations:** 1 Medicine, Avalon University School of Medicine, Willemstad, CUW; 2 Medicine, American University of Barbados, Bridgetown, BRB; 3 Medicine, Aureus University School of Medicine, Oranjestad, ABW; 4 Hospital Medicine, Christine Oxley MD PLLC, Tucson, USA

**Keywords:** public health and safety, hemorrhagic fever, pandemic preparedness, cdc-centre for disease control, marburg virus

## Abstract

Given the recent outbreaks of the Marburg (MARV) virus within the first quarter of the year 2023, interest in the MARV virus has been re-ignited given its shared phylogeny with the dreadful Ebola virus. This relation gives some insight into its virulence, associated morbidities, and mortality rates. The first outbreak of MARV recorded was in Germany, in 1967, of which seven died out of 31 reported cases. Ever since, subsequent cases have been reported all over Africa, a continent replete with failing healthcare systems. This reality impresses a need for a contemporary and concise revision of the MARV virus existing publications especially in the areas of vaccine research. A functional MARV vaccine will serve as a panacea to ailing communities within the African healthcare landscape. The objective of this scoping review is to provide pertinent information relating to MARV vaccine research beginning with an outline of MARV’s pathology and pathogenesis in addition to the related morbidities, existing therapies, established outbreak protocols as well as areas of opportunities.

## Introduction and background

Introduction

Marburg (MARV) virus belongs to the Filoviridae family which is known to cause hemorrhagic fever [1]. Due to the similarities of viruses within the Filoviridae family, scientists have been working on gathering as much information as they can, given the fatal consequences associated with an outbreak [2]. The genus that MARV belongs to is called Marburg virus and contains MARV as well as Ravn virus [3]. MARV was discovered and defined much earlier than its counterparts who share the same family [4]. It has a range of manifestations which include persistent infection, sexual transmission, and long-term sequelae [5-7]. 

MARV has been listed on the Priority Disease List in 2018 [4] given its propensity to cause an outbreak. Since then, this virus has been prioritized for developing countermeasures and preparing adequate responses in case an outbreak does occur. Considerable progress has been made on the vaccine front as well as developing tools necessary for continuing integral research [8]. 

Efforts to continue ongoing vaccine research have been reignited since the recent outbreak in 2023 concerning which the Center for Disease Control (CDC) issued a travel alert against traveling to Equatorial Guinea and Tanzania. The outbreak was declared on February 13, 2023, in Equatorial Guinea with at least nine confirmed cases and seven deaths as well as 20 suspected deaths caused by the virus [9]. Another outbreak was reported in Tanzania on March 21, 2023, with eight cases including five deaths [9]. The Ministry of Health and Social Welfare of Equatorial Guinea stated that the cases initially presented with fever which subsequently led to weakness, vomiting, and bloody diarrhea [5-7]. However, prior outbreaks in Africa have been far more alarming with higher mortality rates [10]. The recent outbreaks, albeit with fewer reported mortalities, have spurred on World Health Organization (WHO) as well as other experts in the field to refocus their efforts on MARV vaccine research.

Materials and methods

Search Strategy

Pubmed and Google Scholar were the search engines used for this scoping review from the start of the study till date of publication. MeSH terms relating to MARV were used and include MARV vaccine, MARV therapies, MARV treatments, MARV, MARV pathology, and pathogenesis to name a few. The websites of the WHO and CDC were consulted for established outbreak protocol using such MeSH terms as virus outbreak and MARV precautions. Sections of the paper were selected by each reviewer and the said reviewer was tasked with conducting independent search and selection of the relevant studies related to the chosen section. The separate body of work was joined together and critiqued by all reviewers for relevance before a final draft was established. All duplicate studies were removed and the lead reviewer made the final edits. 

Study Design and Inclusion Criteria

This scoping review included only MARV vaccine research of which Phase 1 clinical trials had commenced. The selected studies administered a vaccine regimen to healthy participants and measured the strength of protection conferred over weeks to months. Given the limitations in available literature surrounding MARV vaccines, the chosen studies were not assessed for any risk of bias. A comprehensive scoping review was conducted using sources that allowed access to peer-reviewed literature as well as publicly available health guidelines. 

History

MARV was first discovered during a series of outbreaks of hemorrhagic fever in the German cities of Marburg and Frankfurt in 1967; the name of the virus came from the city that had the highest number of infections [2]. A total of 31 patients suffered severe diseases that eventually ended in death for seven of them [11]. The source was tracked back to African green monkeys that were imported from Uganda [5]. Between the first reported outbreak and the early 1990s, only a small number of sporadic MARV infections occurred in contrast with more-widely publicized viruses, which led to the false belief that the MARV was no longer the threat it used to be [5]. 

MARV reintroduced itself with two substantial outbreaks in the Democratic Republic of the Congo (DRC) in 1998-2000 and in Angola (Western Africa) in 2004-2005 [12-13]. The fatality rate was 83% in DRC with 154 total cases and 90% in Angola with 252 total cases that varied in severity [14-15]. Interestingly, it was found that in DRC, there were at least nine different variants circulating, compared to Angola which had one that subsequently spread through person-to-person transmission [12, 16]. The outbreak in DRC was traced back to a gold mine in Durba; for this reason, the outbreak was initially referred to as “hemorrhagic syndrome of Durba'' until it was later confirmed to be a result of the MARV [10]. 

By 2008, there had been 452 total cases reported and 368 confirmed deaths, but experts believe that these numbers are likely higher in reality [10]. According to WHO, there were recent confirmed cases in Guinea in 2021, and Ghana in 2022 and the most recent news reports an outbreak in Equatorial Guinea [9]. Its first confirmed case was on February 12th, 2023, but this is the subject of the ongoing investigation. Per WHO, the Ministry of Health and Social Welfare of Equatorial Guinea has already reported at least nine deaths in a one-month span and five deaths in Tanzania that are related to the MARV [9]. Although the incidence rates of the MARV are not as high as others in the Filoviridae family and those more widely publicized in the news media, its fatality rates remain a concern. Table 1 below lists the known MARV outbreaks in reverse chronological order, with cases vs. deaths and respective fatality rates, based on information from both CDC and WHO. These countries pose a risk to travelers who may encounter the MARV and subsequently spread it to other countries. 

**Table 1:** Chronology of major Marburg (MARV) virus disease outbreaks. The information in the above table was taken from Refs [9, 17-18]. DRC, Democratic Republic of the Congo

Year	Country	Cases	Deaths	Case fatality rate (%)
2023	Equatorial Guinea and Tanzania	29 (Equatorial Guinea) 8 (Tanzania)	27 (Equatorial Guinea) 5 (Tanzania)	Cumulative case fatality rate - 86
2022	Ghana	3	2	67
2021	Guinea	1	1	100
2017	Uganda	3	3	100
2014	Uganda	1	1	100
2012	Uganda	15	4	27
2008	Netherlands (ex-Uganda)	1	1	100
2008	US (ex-Uganda)	1	0	0
2007	Uganda	4	1	25
2004-2005	Angola	252	227	90
1998-2000	DRC	154	128	83
1990	Russia	1	1	100
1987	Kenya	1	1	100
1980	Kenya	2	1	50
1975	South Africa	3	1	33
1967	Germany	31	7	23

## Review

Risk factors

MARV is acquired by humans when they come in close contact with infected fruit bats (*Rousettus aegyptiacus*) or their secretions particularly when visiting the caves or mines in the Sub-Saharan region of Africa [19]. Other animals like non-human primates that come in contact with the secretions of the fruit bat can also get infected and further the transmission chain by acting like intermediate vectors between the fruit bats and humans [20-22]. Those with the highest risk of the MARV are the family members and/or the medical staff that have been in close proximity to the people infected with the virus especially when appropriate prevention and control protocols are not followed. Also, individuals who are working as veterinarians or employees in a laboratory that handles non-human primates from Africa are at risk of being exposed to the MARV [17].

The direct spread of the virus between humans occurs with exposure to bodily fluids like urine, feces, semen, breast milk or amniotic fluid, or the blood of infected people [18]. The indirect spread between humans occurs through items that are shared in households such as clothing, bedding, and utensils that can be contaminated with bodily fluids or improper handling of medical equipment in a healthcare setting [23]. Another significant route of spread occurs via cemeteries that are in direct contact with the body of a deceased individual infected with the MARV as the deceased individual remains virulent as long as virions are present in their bloodstream [18].

Pathogenesis

MARV, like many others in the Filoviridae family, binds to host cells by the use of several attachment surface factors such as glycoprotein (GP) that aids in the binding and entry of the virus [24]. Marburg hemorrhagic fever (MHF), which is the most serious complication in MRV-infected people can be divided into three phases which are the initial phase, followed by the early organ phase, and then the late organ or convalescence phase [25]. As of writing this review article, the reported incubation period of the MARV ranges between 2 and 21 days with an average duration of 5-10 days in humans [26-27]. 

Phase 1

The initial phase begins with flu-like symptoms such as chills, very high fever [usually 39°C-40°C (102°F-104°F)], headache, sore throat, muscle aches, joint pain, and malaise 2-21 days after the initial infection [5, 28-29]. On days 5-7, the intensity of the disease is markedly increased with an appearance of maculopapular rash which spreads from torso to limbs as well as conjunctivitis, continuous fever, and symptoms of systemic hemorrhage which include mucosal bleeding, petechiae, bloody stools, and vomitus and bleeding from venipuncture sites along with other symptoms which included lymphadenopathy, leukopenia, and thrombocytopenia [30-34].

Phase 2

Also known as the early organ phase which lasts from days 5 to 13 after the initial onset of symptoms. Infected individuals may develop conjunctival injection, shortness of breath, viral exanthem, and edema [29]. Patients can develop neurological symptoms such as confusion, agitation, increased sensitivity, and seizures, and may progress to coma [35]. Other organs including the kidney, liver, and pancreas are also affected in this phase which can be seen by increased levels of alanine transaminase (ALT) and aspartate transaminase (AST) along with increased serum creatinine which indicates hepatic and renal damage [5, 36-37].

Phase 3

Also called the late organ phase or convalescent phase. This phase usually has one of two clear outcomes; patients either recover from their illness completely or die due to internal bleeding and multiple organ failure [31, 38]. Patients often suffer from severe metabolic disturbances which include convulsions and severe dehydration; this results in severe negative outcomes on overall patient recovery, and can lead to multiorgan dysfunction as seen by widespread tissue necrosis which first affects the liver and lymphatic system, progressing to affect the pancreas, gonads, adrenal glands, thyroid, kidneys, and skin [39-41]. In some patients, sequelae such as arthritis, conjunctivitis, myalgia, and symptoms of psychosis are reported during and after recovery [31-34]. Abortion is a common consequence of an infection in pregnant women, and infants born to infected mothers usually die [42-44].

 MARV pathophysiology in humans

There are limited clinical studies available for MARV due to the rural conditions as well as the critical phenomenon of most of the MARV outbreaks in Africa, and the collection of laboratory and pathological evidence from individuals infected with the virus has been traditionally insufficient. Our knowledge of clinical symptoms and physical exam findings is derived from the information linked to the first epidemic in Germany, with the South African outbreak and smaller outbreaks in other regions of Africa [10].

MARV primarily affects the phagocytic system which was found during the immunochemical and electron microscopic investigations of a case in Kenya in 1987, which detected MARV antigen and virions in both circulating and associated macrophages [45]. Flow cytometric investigations conducted on macaques that were infected with MARV demonstrated the presence of MARV infection in macrophages [46]. MARV also targets the kidney which manifests as proteinuria in MHF patients [25]. The most important sites where MARV replicates in humans are hepatocytes, adrenal cells, macrophages, islet cells of the pancreas, fibroblast-like cells, and the heart [47].

In MARV infection, the liver has the most distinct histopathological characteristics with hepatocytes demonstrating intracellular eosinophilic and filamentous or ovoid inclusions of the virus [48]. It can be found in periportal zones with associated extensive necrosis to the liver tissue resulting in profound hepatocellular swelling and degeneration along with damage to the reticuloendothelial system resulting in increased liver enzymes [49-50]. MARV was also found in the spleen and lymph nodes and is characterized by depletion of white pulp, perifollicular congestion, and centrifollicular necrosis. Skin involvement in MARV is evident 4-5 days after the initial onset of symptoms, with dermal edema, focal hemorrhage, endothelial swelling, and necrosis [51-52]. In the gastrointestinal tract, MARV inclusions can be found in macrophages and fibroblast-like cells with viruses present in necrotic cells and reticular fibers [45]. Kidneys repeatedly demonstrated evidence of acute tubular necrosis with minimal inflammation [45]. MARV has been detected in semen up to 13 weeks after the onset of the disease [53-54]. It also affects the central nervous system of patients, which manifests as subacute encephalitis with histological evidence of glial nodules and perivascular lymphocytic infiltration [27, 55].

Patients usually have disseminated intravascular coagulation (DIC), along with the overall systemic spread of the virus that leads to the multiorgan failures seen in severe and lethal MHF [56-57].

MARV hemorrhagic fever

MARV primarily targets macrophages and dendritic cells [58-59]. It leads to the cessation of the innate response and disrupts the co-stimulation of lymphocytes resulting in lymphopenia and immunosuppression [60]. Increased levels of pro-inflammatory cytokines, especially IL-6, trigger the coagulation cascade [61-62]. In combination with IL-6, TNF-α can also induce changes in vascular permeability which result in coagulation disorders such as DIC, which is further bolstered by hepatocyte infection leading to reduced synthesis of liver-derived clotting factors [63-64]. Decreased levels of protein C, protein S, and fibrinogen are also observed [65].

MARV impairs the adrenocortical cells in the adrenal gland, thereby resulting in the impaired release of steroid-synthesizing enzyme production and release which causes hypovolemia and hypotension, leading to shock [56].

Clinically significant hemorrhage has been observed among MARV-infected individuals at both the macroscopic (e.g., oral and nasal mucosa, GI tract, venipuncture sites, and conjunctiva) and microscopic (e.g., gastric mucosa, ventricular myocardium, interstitial tissue of the testes and lymph nodes) level [66]. During the Democratic Republic of the Congo (DRC) outbreak, clinically apparent hemorrhage was more commonly observed in fatal cases (phase 3 of MHF) than in survivors but these differences were not statistically significant [12].

Clinical presentation

MARV disease (MVD) cannot be clinically diagnosed with high certainty and this creates a unique difficulty when it comes to early detection [67]. The signs and symptoms associated with the disease are ubiquitous when compared to other infectious agents of equal virulence or similar etiology [67]. This inextricably complicates a clinical diagnosis of the disease. It has an incubation period of 2-21 days [67-69]. The signs and symptoms range from mild to severe as the disease progresses. Mild cases are characterized by elevated body temperature, rigors, headache, pharyngitis, chest pain, nausea, vomiting, abdominal pain, diarrhea, and myalgia which appear within the first five days following the incubation period [67-69]. As the disease progresses, a variety of more severe symptoms of end-organ failure become apparent such as jaundice, altered mental status, hypovolemic shock, severe weight loss, and hemorrhage, a hallmark of the disease [67-69]. These symptoms usually become apparent after day 5 of symptom onset [67-69]. The most severe manifestations such as DIC and death can be expected between days 8 and 16 of symptom onset [68]. It is incorrectly assumed that hemorrhage is the cause of death as well as the most severe manifestations of the disease, however, this could not be further from the truth [68]. The severe symptoms and death are a result of a combination of events, namely, hypotension due to fluid redistribution which creates an environment that facilitates widespread intravascular coagulation and tissue hypoxia leading to death [68]. Such manifestations include exanthems such as petechiae, purpura, ecchymoses, as well as hematomas [68].

Management and treatment

At this time, no treatment currently exists for MVD. Management primarily involves addressing the symptoms and providing supportive care in response to metabolic derangements, to prevent further complications or death. After exposure to the virus, it is crucial to manage pain if reported, replenish fluids and electrolytes to prevent dehydration, stabilize oxygen levels and blood pressure, replace blood or clotting factors in the case of hemorrhaging, and treat any secondary infections or complications [17].

Preventative measures and recommendations

MARV can be transmitted from its natural host, the Egyptian fruit bat (Rousettus aegyptiacus), to both human and non-human primates [2]. Infected fruit bats do not show any signs or symptoms of an infection and this makes it a challenging task to interact with such animals [70]. It is highly recommended to completely avoid fruit bats and their habitats as a precaution [71]. In contrast, infection of primates results in hemorrhagic fever [71]. The disease known as MHF can present with a multitude of signs and symptoms, and eventually kills the primate [68, 70-71]. Infected primates (both human and non-human) who are symptomatic are easier to identify and avoid, which reduces the likelihood of transmission through this method [67, 69]. Humans may acquire the virus through exposure to infected fruit bats, infected primates (both human and non-human), or exposure to materials contaminated with bodily fluids such as soiled clothing [70-71]. 

Primary transmission of the virus from fruit bats and non-human primates can be greatly limited or eliminated by reducing direct contact with these animals [68]. Secondary transmission between humans can be further mitigated by following proper safety, sterilization, and disposal protocols [67, 70]. An encounter with an infected human must follow measures to prevent transmission such as establishing a quarantine zone for strict isolation of the patient; eliminating non-essential contact; wearing protective gowns, gloves, and masks when interacting with a patient; proper sterilization of all re-usable equipment that comes in contact with the patient or area of isolation; proper disposal of patient waste; and using disposable items such as utensils, cups, toothbrushes, and clothing [68, 70].

In addition to the aforementioned measures, proper education of communities of both healthcare and non-healthcare personnel will improve awareness and stop the spread of the virus [70-71]. Such measures will ensure stronger precautions are created against the spread of the virus within a community, more specifically among grieving family members and healthcare providers [70-71]. Also, given the advances in technology, improving the accessibility of diagnostic tools to remote areas should increase the possibility of early detection, isolation, and treatment. Development and distribution of rapid testing kits is highly recommended. Lastly, vaccination of individuals in endemic areas is of utmost importance in curbing the morbidity and mortality of the disease; however, at the time of writing, no vaccine for the MARV has yet been approved [72].

Vaccines and research

As of this writing, no vaccine or specific therapy (aside from supportive care) has been approved worldwide or specific therapy for MARV disease. Many MARV candidate platforms for non-human primates (NHPs) have been demonstrated to provide protection against viruses such as recombinant vesicular stomatitis virus (rVSV), virus-like particles (VLPs), and adenovirus (AdVs) [73]. The three MARV vaccines with the highest potential for successful immunity are Chimpanzee adenovirus serotype 3 vector (cAd3), modified vaccinia Ankara vector (MVA-BN-Filo), and MARV DNA plasmid vaccine (VRC-MARDNA025-00-VP) [72]. These vaccines are in Phase 1 clinical trials except the MVA-BN-Filo is scheduled for Phase 2/3 clinical trials [2]. 

Vaccines using DNA vectors are promising, but their comparative efficacy as a stand-alone platform has been limited in primates due to suboptimal gene expression or cell targeting [74-76]. A MARV DNA plasmid vaccine (VRC-MARDNA025-00-VP) expressing MARV Angola DNA has completed Phase 1 clinical testing. A vaccine was distributed among 10 volunteers at 0, 4, and 8 weeks and 90% had antibody responses. Seven out of 10 received a fourth dose at 12 weeks, which boosted diminishing antibody titers. Currently, there are no Phase 2/3 trials taking place [2]. 

Since 1967, there have been only 16 outbreaks of MVD, including that involving over 100 cases. Most outbreaks are relatively small and need to be controlled quickly using existing infection control and public health measures. Therefore, it is suggested that multiple outbreaks could be used to conduct Phase 3 trials for MVD vaccines until sufficient endpoints have been accumulated to assess vaccine efficacy (VE) and can be estimated by estimating the number of outbreaks required [77]. 

Several adenovirus-based vaccine studies are limited to MARV. Phase 1 clinical trials have demonstrated that an experimental MARV vaccine called the cAd3 Marburg vaccine displays a GP found on the surface of MARV to induce immune responses [78]. Clinical trials using the cAd3 vaccine demonstrated a good safety proﬁle. However, the pre-existing immunity against the adenovirus in the population has limited the use of adenovirus vectors [72, 79]. Animal models have been used to alleviate this problem by using less common serotypes in animal vaccination and by administering the vaccines either orally or nasally. Currently, there are no Phase 2/3 trials taking place [80].

In 2016 a Phase 1 clinical trial was published where 87 participants were randomized to receive either Zabdeno (Ad26.ZEBOV-GP, recombinant) or the MVA-BN-Filo vaccine, encoding GPs common among viruses found within the same phylogeny as MARV [72, 81]. Following primary immunization, the subjects receive an alternate shot and are boosted with the new vaccine at 14, 28, or 56 days after primary immunization [82]. There were no serious adverse reactions associated with the vaccine. There was a detectable IgG response in 97% of Ad26.ZEBOV recipients after 28 days of immunization, and in 23% of MVA-BN-Filo recipients after 21 days and 8 months after receiving the alternate vaccine [82]. Phase 2/3 trials will be soon conducted on both vaccines [81-83]. Another study conducted in Rwanda between 2019 and 2021, showed no adverse effects from the MVA-BN-Filo vaccine which was trialled on a population of over 200,000 people [81].

The WHO states that the odds are against a successful trial because the necessary measures to combat an outbreak (e.g. quarantine) could potentially end the outbreak before a single vaccine can be administered. Even if a trial has commenced, there may be an inadequate number of cases to accurately judge the vaccine’s effectiveness. Evidence points to creating an effective vaccine by gathering information from multiple outbreaks [83]. 

Discussion

The recent COVID-19 pandemic has brought all the potential pandemic-causing pathogens to the limelight. Even though the epidemiological spread of respiratory viruses is much easier than contact-spread viruses like the viruses of the Filoviridae family, the high mortality rate of these viruses always draws focus and cannot be ignored. The average mortality percentage of the MARV infection is estimated to be about 50%, compared to the estimated mortality rate of the SARS-CoV-2 (Covid-19) virus at only 0.5%-2% [18, 84]. Soaring globalization in the world today has made it increasingly difficult to contain, trace, and manage outbreaks that might otherwise be endemic to a region. It also makes countries with weaker economies particularly vulnerable to such outbreaks which can lead to inadequate containment and management of the pathogenic spread and may prolong the outbreak.

Only a handful of MARV outbreaks have been reported but the devastation it has caused has been enormous. Although infected fruit bats act as the primary host, the virus has been reportedly found in other animals like pigs and nonhuman primates furthering the transmission chain [18]. Hence, it is important to understand the reservoir-host interactions with other animals and the host-to-human transmission cycle to come up with decisive strategies and policies that can not only help contain the spread but also help us develop novel therapeutic drugs, and possible vaccines, and come up with better diagnostic methods. Regular farm checkups of the animals, proper culling and burying of the infected animals, and reducing interaction with the primary hosts, the fruit bats should be some of the strategies the governments can adopt into policy to curtail future outbreaks.

Foremost importance has to be given to improving awareness of MVD among the general population of the endemic areas as well as healthcare personnel. It is crucial that they are educated not only about the modes of viral transmission but also on how to identify the early symptoms of MVD. This ensures early identification and isolation of the infected individuals and may help contain the outbreak. Moreover, definitive government policies and protocols need to be forged to minimize or prevent human-to-human transmission, with particular attention on limiting exposure to bodily fluids. This can include hygienic and dignified burial practices, safer sexual practices, and environmental hygiene. Nosocomial spread of the virus must be taken extremely seriously and is necessary to limit the interaction of healthcare personnel with the infected individual. Furthermore, all healthcare personnel have to be educated about self-protective practices, respiratory hygiene, safe injection practices, proper hand washing techniques as well as proper contaminated waste disposal techniques in order to protect themselves and other patients in the hospital [2]. 

Deeply rooted cultural or traditional practices and lack of healthcare education amongst the masses can sometimes lead to people acquiring the virus. An example of this is unsafe cultural burying practices in the endemic regions of Africa which may cause significant exposure to the dead bodies who fell victim to the MARV [85]. The government has to work with local and regionally influential people like religious leaders, local politicians, and healthcare personnel to improve awareness about harmful cultural practices and help people find more hygienic and safe ways of life without hindering the cultural importance of the region.

The MARV disease has no known cure or approved vaccine; hence, all the research is limited to only a handful of highly fortified laboratories around the world. Various types of vaccines are in different stages of development but novel vaccine platforms like the viral vector vaccine and DNA vaccine have shown protective efficacy in rodents and nonhuman primates [86]. The most vulnerable population must be identified and prioritized when administering the vaccine, such as research laboratory personnel, people at risk of occupational exposure, like miners, and people in endemic areas where the virus has been known to cause outbreaks. 

Effortless exchange of information about the virus between governments and multinational organizations like WHO is crucial. This will not only help other countries screen their own people but also ensure the victim countries receive international help as soon as possible. International protocols and regulations must be set up in order to be prepared for an anticipated outbreak. Governments around the world along with the UN and WHO must bring about policies for pandemic education, containment measures, and of healthcare personnel training. Anticipating an outbreak and having the infrastructure and resources ready, and trained manpower to overcome is the best strategy/blueprint for the future.

## Conclusions

Recurrent outbreaks and the high mortality risk of the Filoviridae family of viruses make it very essential to continue MARV vaccine research. Concrete governmental policies are needed to limit the exposure of people to zoonotic hosts and minimize human-to-human transmission if an outbreak is confirmed. It is crucial to increase awareness and education in the general population about the animal hosts of the virus, viral transmission, and precautionary measures that need to be taken to prevent the transmission of the virus. Healthcare personnel must strictly adhere to the regulations and protocols set up by governmental and international agencies like WHO to curb any possible nosocomial spread. It is the need of the hour for extensive research into developing a cure or a vaccine that can potentially save hundreds if not thousands of lives in the future. 

## References

[1] Atkins C, Miao J, Kalveram B (2018). Natural history and pathogenesis of wild-type Marburg virus infection in STAT2 knockout hamsters. J Infect Dis.

[2] Kortepeter MG, Dierberg K, Shenoy ES (2020). Marburg virus disease: a summary for clinicians. Int J Infect Dis.

[3] Kuhn JH, Bao Y, Bavari S (2013). Virus nomenclature below the species level: a standardized nomenclature for natural variants of viruses assigned to the family Filoviridae. Arch Virol.

[4] Olejnik J, Mühlberger E, Hume AJ (2019). Recent advances in marburgvirus research. F1000Res.

[5] Gear JS, Cassel GA, Gear AJ (1975). Outbreake of Marburg virus disease in Johannesburg. Br Med J.

[6] Smith DH, Johnson BK, Isaacson M (1982). Marburg-virus disease in Kenya. Lancet.

[7] Johnson ED, Johnson BK, Silverstein D (1996). Characterization of a new Marburg virus isolated from a 1987 fatal case in Kenya. Arch Virol Suppl.

[8] Connor J, Kobinger G, Olinger G (2017). Therapeutics against filovirus infection. Curr Top Microbiol Immunol.

[9] Marburg virus disease - Equatorial Guinea (2023 (2023). Marburg virus disease - Equatorial Guinea. https://www.who.int/emergencies/disease-outbreak-news/item/2023-DON449.

[10] Brauburger K, Hume AJ, Mühlberger E (2012). Forty-five years of Marburg virus research. Viruses.

[11] Slenczka W, Klenk HD (2007). Forty years of Marburg virus. J Infect Dis.

[12] Bausch DG, Nichol ST, Muyembe-Tamfum JJ (2006). Marburg hemorrhagic fever associated with multiple genetic lineages of virus. N Engl J Med.

[13] Qiu X, Wong G, Audet J (2014). Establishment and characterization of a lethal mouse model for the Angola strain of Marburg virus. J Virol.

[14] Bausch DG, Feldmann H, Geisbert TW (2007). Outbreaks of filovirus hemorrhagic fever: time to refocus on the patient. J Infect Dis.

[15] Towner JS, Khristova ML, Sealy TK (2006). Marburgvirus genomics and association with a large hemorrhagic fever outbreak in Angola. J Virol.

[16] Bertherat E, Talarmin A, Zeller H (1999). [Democratic Republic of the Congo: between civil war and the Marburg virus. International Committee of Technical and Scientific Coordination of the Durba Epidemic]. Medecine Tropicale : Revue du Corps de Sante Colonial.

[17] Marburg disease outbreaks (2023 (2023). Marburg virus disease outbreaks. Centers for Disease Control and Prevention. https://www.cdc.gov/vhf/marburg/outbreaks/chronology.html.

[18] (2023). Marburg virus disease World Health Organization. https://www.who.int/news-room/fact-sheets/detail/marburg-virus-disease.

[19] Nikiforov VV, Turovskii Iu I, Kalinin PP (1994). A case of a laboratory infection with Marburg fever (Article in Russian). Zh Mikrobiol Epidemiol Immunobiol.

[20] Martini GA (1973). Marburg virus disease. Postgrad Med J.

[21] Siegert R, Shu HL, Slenczka HL (1968). The aetiology of an unknown human infection transmitted by monkeys (preliminary communication). Ger Med Mon.

[22] Leroy EM, Rouquet P, Formenty P (2004). Multiple Ebola virus transmission events and rapid decline of central African wildlife. Science.

[23] Schwartz DA (2019). Maternal filovirus infection and death from Marburg and Ravn viruses: highly lethal to pregnant women and their fetuses similar to Ebola virus. IntechOpen.

[24] Hoffmann M, Crone L, Dietzel E (2017). A polymorphism within the internal fusion loop of the Ebola virus glycoprotein modulates host cell entry. J Virol.

[25] Martini GA (1971). Marburg Virus Disease. Clinical Syndrome. Berlin,Heidelberg: Springer Berlin Heidelberg.

[26] Pavlin BI (2014). Calculation of incubation period and serial interval from multiple outbreaks of Marburg virus disease. BMC Res Notes.

[27] Ewers EC, Pratt WD, Twenhafel NA (2016). Natural history of aerosol exposure with Marburg virus in Rhesus Macaques. Viruses.

[28] Martini GA, Knauff HG, Schmidt HA (1968). On the hitherto unknown, in monkeys originating infectious disease: Marburg virus disease (Article in German). Dtsch Med Wochenschr.

[29] Klenk HD, Slenczka W, Feldmann H (1999). Marburg and Ebola viruses (Filoviridae). J Infect Dis.

[30] Borchert M, Mulangu S, Lefevre P (2007). Use of protective gear and the occurrence of occupational Marburg hemorrhagic fever in health workers from Watsa health zone, Democratic Republic of the Congo. J Infect Dis.

[31] Kuhn JH (2008). Filoviruses: a compendium of 40 years of epidemiological, clinical, and laboratory studies. Arch Virol Suppl.

[32] Hartman AL, Towner JS, Nichol ST (2010). Ebola and Marburg hemorrhagic fever. Clin Lab Med.

[33] Feldmann H (2006). Marburg hemorrhagic fever--the forgotten cousin strikes. N Engl J Med.

[34] Leroy E (2009). Chapter 199 - filoviral hemorrhagic fever: Marburg and ebola virus fevers. Feigin and Cherry’s Textbook of Pediatric Infectious Diseases, 6th ed..

[35] Borchert M, Muyembe-Tamfum JJ, Colebunders R (2002). Short communication: a cluster of Marburg virus disease involving an infant. Trop Med Int Health.

[36] Richards GA, Murphy S, Jobson R (2000). Unexpected Ebola virus in a tertiary setting: clinical and epidemiologic aspects. Crit Care Med.

[37] Paweska JT, Jansen van Vuren P, Fenton KA (2015). Lack of Marburg virus transmission from experimentally infected to susceptible in-contact Egyptian fruit bats. J Infect Dis.

[38] Borchert M, Van der Stuyft P (2009). Epidemiology and control of Marburg haemorrhagic fever epidemics in Central Africa. Afrika Focus.

[39] Gedigk P, Bechtelsheimer H, Korb G (1968). [Pathological anatomy of the "Marburg virus" disease (the so-called "Marburg monkey disease")]. Dtsch Med Wochenschr.

[40] Peters CJ, Zaki SR (2002). Role of the endothelium in viral hemorrhagic fevers. Crit Care Med.

[41] Zaki SR, Goldsmith CS (1999). Pathologic features of filovirus infections in humans. Curr Top Microbiol Immunol.

[42] Schwartz DA (2018). Clinical trials and administration of Zika virus vaccine in pregnant women: lessons (that should have been) learned from excluding immunization with the Ebola vaccine during pregnancy and lactation. Vaccines (Basel).

[43] Bebell LM, Riley LE (2015). Ebola virus disease and Marburg disease in pregnancy: a review and management considerations for filovirus infection. Obstet Gynecol.

[44] Bebell LM (2019). Ebola virus disease and pregnancy: perinatal disease and transmission. Pregnant in the Time of Ebola: Women and Their Children in the 2013-2015 West African Epidemic.

[45] Geisbert TW, Jaax NK (1998). Marburg hemorrhagic fever: report of a case studied by immunohistochemistry and electron microscopy. Ultrastruct Pathol.

[46] Fritz EA, Geisbert JB, Geisbert TW (2008). Cellular immune response to Marburg virus infection in cynomolgus macaques. Viral Immunol.

[47] Connolly BM, Steele KE, Davis KJ (1999). Pathogenesis of experimental Ebola virus infection in guinea pigs. J Infect Dis.

[48] Murphy FA (1978). Pathology of Ebola virus infection. Ebola Virus Hemorrhagic Fever.

[49] Geisbert TW, Daddario-DiCaprio KM, Geisbert JB (2007). Marburg virus Angola infection of rhesus macaques: pathogenesis and treatment with recombinant nematode anticoagulant protein c2. J Infect Dis.

[50] Kortepeter MG, Bausch DG, Bray M (2011). Basic clinical and laboratory features of filoviral hemorrhagic fever. J Infect Dis.

[51] Nkoghe D, Leroy EM, Toung-Mve M (2012). Cutaneous manifestations of filovirus infections. Int J Dermatol.

[52] Zaki SR, Shieh WJ, Greer PW (1999). A novel immunohistochemical assay for the detection of Ebola virus in skin: implications for diagnosis, spread, and surveillance of Ebola hemorrhagic fever. Commission de Lutte contre les Epidémies à Kikwit. J Infect Dis.

[53] Bausch DG, Towner JS, Dowell SF (2007). Assessment of the risk of Ebola virus transmission from bodily fluids and fomites. J Infect Dis.

[54] Martini GA, Schmidt HA (1968). Spermatogenic transmission of the "Marburg virus". (Causes of "Marburg simian disease") (Article in German). Klin Wochenschr.

[55] Bechtelsheimer H, Korb G, Gedigk P (1970). The 'Marburg-virus'-hepatitis. Studies in man and guinea pigs. Virchows Arch A Pathol Pathol Anat.

[56] Geisbert TW (2014). 166 - Marburg and Ebola hemorrhagic fevers (Filoviruses). Mandell, Douglas, and Bennett's Principles and Practice of Infectious Diseases.

[57] Havemann K, Schmidt HA (1971). Haematological findings in Marburg virus disease: evidence for involvement of the immunological system. Marburg Virus Disease.

[58] Reis e Sousa C (2004). Toll-like receptors and dendritic cells: for whom the bug tolls. Semin Immunol.

[59] Reis e Sousa C (2004). Activation of dendritic cells: translating innate into adaptive immunity. Curr Opin Immunol.

[60] Baize S, Leroy EM, Georges-Courbot MC (1999). Defective humoral responses and extensive intravascular apoptosis are associated with fatal outcome in Ebola virus-infected patients. Nat Med.

[61] Hensley LE, Geisbert TW (2005). The contribution of the endothelium to the development of coagulation disorders that characterize Ebola hemorrhagic fever in primates. Thromb Haemost.

[62] Leroy EM, Baize S, Volchkov VE (2000). Human asymptomatic Ebola infection and strong inflammatory response. Lancet.

[63] Ströher U, West E, Bugany H (2001). Infection and activation of monocytes by Marburg and Ebola viruses. J Virol.

[64] Feldmann H, Bugany H, Mahner F (1996). Filovirus-induced endothelial leakage triggered by infected monocytes/macrophages. J Virol.

[65] Ebihara H, Rockx B, Marzi A (2011). Host response dynamics following lethal infection of rhesus macaques with Zaire ebolavirus. J Infect Dis.

[66] Egbring R, Slenczka W, Baltzer G (1971). Clinical manifestations and mechanism of the haemorrhagic diathesis in Marburg virus disease. Marburg Virus Disease.

[67] Marburg disease outbreaks (2023 (2023). Marburg disease outbreaks (2023). Centers for Disease Control and Prevention. https://www.cdc.gov/vhf/marburg/symptoms/index.html.

[68] Mehedi M, Groseth A, Feldmann H (2011). Clinical aspects of Marburg hemorrhagic fever. Future Virol.

[69] (2023). Marburg virus disease. World Health Organization. https://www.who.int/health-topics/marburg-virus-disease/#tab=tab_2.

[70] Islam MR, Akash S, Rahman MM (2023). Epidemiology, pathophysiology, transmission, genomic structure, treatment, and future perspectives of the novel Marburg virus outbreak. Int J Surg.

[71] Vega-Rodriguez W, Ly H (2023). Emergence of deadly viral haemorrhagic fever disease outbreaks in West Africa. Virulence.

[72] Dulin N, Spanier A, Merino K (2021). Systematic review of Marburg virus vaccine nonhuman primate studies and human clinical trials. Vaccine.

[73] Reynolds P, Marzi A (2017). Ebola and Marburg virus vaccines. Virus Genes.

[74] Lu S, Wang S, Grimes-Serrano JM (2008). Current progress of DNA vaccine studies in humans. Expert Rev Vaccines.

[75] Falzarano D, Geisbert TW, Feldmann H (2011). Progress in filovirus vaccine development: evaluating the potential for clinical use. Expert Rev Vaccines.

[76] Martin JE, Sullivan NJ, Enama ME (2006). A DNA vaccine for Ebola virus is safe and immunogenic in a phase I clinical trial. Clin Vaccine Immunol.

[77] Qian GY, Jombart T, Edmunds WJ (2023). Qian GY, Jombart T, Edmunds WJ. Assessing the feasibility of Phase 3 vaccine trials against Marburg Virus Disease: a modelling study. https://doi.org/10.1101/2023.02.22.23286294.

[78] Geisbert TW, Bailey M, Geisbert JB (2010). Vector choice determines immunogenicity and potency of genetic vaccines against Angola Marburg virus in nonhuman primates. J Virol.

[79] Swenson DL, Wang D, Luo M (2008). Vaccine to confer to nonhuman primates complete protection against multistrain Ebola and Marburg virus infections. Clin Vaccine Immunol.

[80] Long MJ, Aye Y (2021). Science's response to CoVID-19. ChemMedChem.

[81] Nyombayire J, Ingabire R, Magod B (2023). Monitoring of adverse events in recipients of the 2-dose Ebola vaccine regimen of Ad26.ZEBOV followed by MVA-BN-Filo in the UMURINZI Ebola vaccination campaign. J Infect Dis.

[82] Milligan ID, Gibani MM, Sewell R (2016). Safety and immunogenicity of novel adenovirus type 26- and modified vaccinia Ankara-vectored Ebola vaccines: a randomized clinical trial. JAMA.

[83] Callaway E (2023). Marburg virus outbreak: researchers race to test vaccines. Nature.

[84] Stanley DA, Honko AN, Asiedu C (2014). Chimpanzee adenovirus vaccine generates acute and durable protective immunity against ebolavirus challenge. Nat Med.

[85] Ligon BL (2005). Outbreak of Marburg hemorrhagic fever in Angola: a review of the history of the disease and its biological aspects. Semin Pediatr Infect Dis.

[86] Suschak JJ, Schmaljohn CS (2019). Vaccines against Ebola virus and Marburg virus: recent advances and promising candidates. Hum Vaccin Immunother.

